# Comparing the Performance of Cluster Random Sampling and Integrated Threshold Mapping for Targeting Trachoma Control, Using Computer Simulation

**DOI:** 10.1371/journal.pntd.0002389

**Published:** 2013-08-22

**Authors:** Jennifer L. Smith, Hugh J. W. Sturrock, Casey Olives, Anthony W. Solomon, Simon J. Brooker

**Affiliations:** 1 London School of Hygiene and Tropical Medicine, London, United Kingdom; 2 London Centre for Neglected Tropical Diseases Research, London, United Kingdom; 3 Global Health Group, University of California San Francisco, San Francisco, California, United States of America; 4 University of Washington, Seattle, Washington, United States of America; 5 Kenya Medical Research Institute-Wellcome Trust Research Programme, Nairobi, Kenya; University of California San Francisco, United States of America

## Abstract

**Background:**

Implementation of trachoma control strategies requires reliable district-level estimates of trachomatous inflammation–follicular (TF), generally collected using the recommended gold-standard cluster randomized surveys (CRS). Integrated Threshold Mapping (ITM) has been proposed as an integrated and cost-effective means of rapidly surveying trachoma in order to classify districts according to treatment thresholds. ITM differs from CRS in a number of important ways, including the use of a school-based sampling platform for children aged 1–9 and a different age distribution of participants. This study uses computerised sampling simulations to compare the performance of these survey designs and evaluate the impact of varying key parameters.

**Methodology/Principal Findings:**

Realistic pseudo gold standard data for 100 districts were generated that maintained the relative risk of disease between important sub-groups and incorporated empirical estimates of disease clustering at the household, village and district level. To simulate the different sampling approaches, 20 clusters were selected from each district, with individuals sampled according to the protocol for ITM and CRS. Results showed that ITM generally under-estimated the true prevalence of TF over a range of epidemiological settings and introduced more district misclassification according to treatment thresholds than did CRS. However, the extent of underestimation and resulting misclassification was found to be dependent on three main factors: (i) the district prevalence of TF; (ii) the relative risk of TF between enrolled and non-enrolled children within clusters; and (iii) the enrollment rate in schools.

**Conclusions/Significance:**

Although in some contexts the two methodologies may be equivalent, ITM can introduce a bias-dependent shift as prevalence of TF increases, resulting in a greater risk of misclassification around treatment thresholds. In addition to strengthening the evidence base around choice of trachoma survey methodologies, this study illustrates the use of a simulated approach in addressing operational research questions for trachoma but also other NTDs.

## Author Summary

Reliable district-level prevalence estimates of active trachoma are essential to targeting control interventions. While cluster randomised surveys (CRS) remain the recommended strategy for obtaining these estimates, more rapid and cost-effective methods that can be integrated with other diseases are under investigation. One proposed method is Integrated Threshold Mapping (ITM), which incorporates a school-based platform into the sampling protocol. This study uses a computerised sampling approach to evaluate whether ITM and CRS are equivalent, and explore the impact of varying key parameters on the performance of these sampling methodologies. The results from these simulations reflect a known limitation of school-based sampling: that resulting prevalence estimates are unreliable when the enrollment is low and/or the risk of disease in schools differs from communities. However, quantification of the performance of ITM at the district level highlights the variation in performance in different contexts and provides important information for national control programmes. The results from this study strengthen the evidence base around trachoma sampling methodologies and demonstrate the advantages of using a simulated approach to evaluate different sampling scenarios.

## Introduction

Since the establishment in 1998 of the Global Elimination of Trachoma by 2020 (GET2020) Alliance, an increasing number of endemic countries have implemented national programmes in an effort to meet elimination targets. These targets are less than one case of trachomatous trichiasis (TT) per 1000 total population unknown to the health system, and <5% trachomatous inflammation–follicular (TF) in children aged 1–9 years, at the sub-district level [1]. In response to these targets and a need to finalise global mapping in time to allow programmatic impact, there has been a renewed interest in developing cost-effective mapping strategies and integrating survey and control activities with other neglected tropical diseases (NTDs) [2]–[5]. Population-based prevalence surveys (PBPS) remain the accepted “gold standard” for estimating the prevalence of trachoma within target populations and usually use cluster random sampling (CRS) to select non-overlapping subpopulations (clusters) [6]. This methodology is relatively expensive, however, and there is interest in developing cheaper and more rapid methods as well as integrating with other disease surveys [7]. Integrated Threshold Mapping (ITM) is a sampling methodology currently being put forward as a cost-effective means of rapidly surveying trachoma in remaining unmapped districts and to allow treatment decisions to be made and timely scale up of interventions to be achieved [8].

Both CRS and ITM diagnose trachoma based on the presence of key clinical signs using the 1987 WHO simplified grading system: TF in children aged 1–9 and TT in adults aged over 14 [9]. These measures are easily collected in the field and routinely used to inform intervention strategies. For example, in districts where the prevalence of TF is greater than 10%, annual mass drug administration (MDA) of azithromycin should be implemented (Table 1). However, ITM differs from the accepted “gold standard” survey methodology in a number of important ways, briefly outlined in Table 2, including the use of a school-based sampling platform for children aged 1–9 and a different age distribution of participants. Differences in selection of participants can have a varying impact on resulting prevalence estimates and treatment decisions, depending on how disease is distributed in the population. Age patterns of active trachoma indicate a higher burden in children under 10 years, with the highest prevalences found in preschool-aged children in hyperendemic areas [10], [11]. A recent meta-analysis has reported the risk of TF to be lower in children attending school in four African countries [12], supporting widely-held beliefs that the risk of trachoma is likely to vary by attendance (and enrollment) in trachoma endemic contexts.. While CRS takes a community-based sample, that theoretically is representative of the true age distribution and prevalence of disease in this population, ITM may over- or under-sample certain age groups and introduce a bias if the risk differs between enrolled and non-enrolled children. In addition, clustering of active trachoma by household has been observed in a number of studies [13]–[15], and the precision of estimates from both sampling methodologies are expected to be influenced by this factor. A careful evaluation of how participant selection and variation in epidemiological parameters impact prevalence estimates and treatment decisions using the two methodologies is warranted.

**Table 1:** Azithromycin treatment strategies and classification at designated TF prevalence thresholds [1].

TF Prevalence (district level)	Classification	Treatment strategy
<5%	Active trachoma not a public health problem	No MDA
5–9.9%	Hypo-endemic	Determine need for MDA at sub-district level
10–29.9%	Meso-endemic	MDA at district level (≥3 years^a^)
>30%	Hyper-endemic	MDA at district level (≥5 years^a^)

a before reassessment to determine whether to stop or continue.

**Table 2:** Methodological differences between cluster random sampling (CRS) and Integrated Threshold Mapping (ITM).

	CRS	ITM
Platform	Community-based	School-based with younger children brought from the community
Cluster selection	Probability proportional to size or random selection	Random selection: minimum 2 per subdistrict
Participant selection	Household	Children aged 6–9 at school & 1–5 year old children from communities
Sample size and age groups	100 aged 1–9 years	25 aged 1–5 years and 25 aged 6–9 years

Although ITM was internally validated against CRS during the pilot phase of the methodology's development in Mali and Senegal [8], and used in a nationwide mapping of Togo [16], these evaluations were limited by several issues. In Mali and Senegal, only a single district was surveyed providing limited evidence in trachoma meso- and hyperendemic settings. Furthermore, the CRS sample in these settings was partially comprised of existing ITM clusters, which could potentially have biased the CRS estimates and resulted in an overly-optimistic assessment of ITM. Finally, although this methodology was used to map trachoma in all districts in Togo, it is a trachoma hypoendemic country and so results could not be generalised to other trachoma endemic contexts.

Computerised sampling simulations have provided a convenient platform recently to evaluate alternative survey designs for tropical diseases including soil-transmitted helminthes, trachoma and schistosomiasis [17]–[20]. This approach entails generating realistic “gold standard” data for a population that maintains observed disease clustering, using epidemiological parameters derived from existing datasets. A survey methodology can then be evaluated using these data by selecting participants according to the specified sampling protocol and deriving a prevalence estimate. There are a number of advantages to using computerized sampling simulations to compare survey designs, including the ability to i) simulate fully enumerated data (allowing estimation of “true” prevalence of disease), ii) incorporate sampling error by repeating simulations a large number of times, iii) evaluate performance across a range of endemicity settings and iv) explore how variation in factors underlying clustering of disease in communities will influence the performance of sampling methodologies. A similar comparison performed empirically might be prohibitively expensive to carry out, as it would require at minimum a full census survey of a large number of districts across different endemicity settings and implementation of each sampling protocol in the field.

This analysis used computerised sampling simulations to compare the precision and accuracy of district level prevalence estimates based on ITM versus CRS. Furthermore, we compared the performance of both survey methodologies, in terms of their ability to correctly classify districts according to established TF prevalence thresholds and the factors that affect the degree of equivalence. Equivalence between the two survey methods, under different scenarios, was formally evaluated by testing the null hypothesis that ITM yields the same programmatic results compared to CRS.

## Materials and Methods

Simulating sampling designs require gold standard data from which to draw samples and compare sample estimates. There are no perfect datasets available to conduct this analysis, which would necessitate standarised, full census datasets of demographic and epidemiological information for multiple districts. An alternative is to simulate these data, using parameter estimates from empirical data to generate realistic pseudo gold standard data on active trachoma [21], [22]. In this study, full census data from a single community are used to parameterize disease clustering and, incorporating information on between-district variation, to ‘expand’ the available dataset and generate data for a large number of simulated communities within many districts.

### Empirical datasets

#### Community level dataset

One dataset used to parameterize this analysis comes from Kahe Village, Rombo District, northern Tanzania, which is a single community that consists of 90 local administrative units called balozis. A fully enumerated census and survey of trachoma was conducted in April to June 2000 by means of a house-to-house survey, using the WHO simplified grading system, prior to the initiation of any interventions against trachoma. A single examiner collected these data and clinical grading was validated through a live-patient inter-grader agreement exercise using an international expert reference grader with an agreement of 100% for TF. The dataset in total consists of 5748 individuals in 1103 households, with between 41–126 individuals and 8–23 households per balozi. The dataset included information on the presence or absence of TF in 1831 children aged 1–9 years, where the prevalence was 33.4%. Data on school enrolment were also available for a subset (23%) of children aged 6–9 years.

The demographic (age and gender) and household structure present in Kahe was used for all simulated communities in the expanded dataset. This dataset was also used to provide initial values used to parameterize the models, including the relative risk of TF between children aged 1–5 years and 6–9 years and the intra-cluster correlation (ICC) measuring the degree of disease clustering within households. The subset of data with information on enrolment provided an initial value for the relative risk of TF in children aged 6–9 who were enrolled in school to those who did not. In addition, this dataset was used to assess whether there was an additional household level risk associated with having a schoolgoing/non-schoolgoing sibling and inform the simulation model (results presented in the Technical Appendix).

#### District level dataset

**Figure 1:**
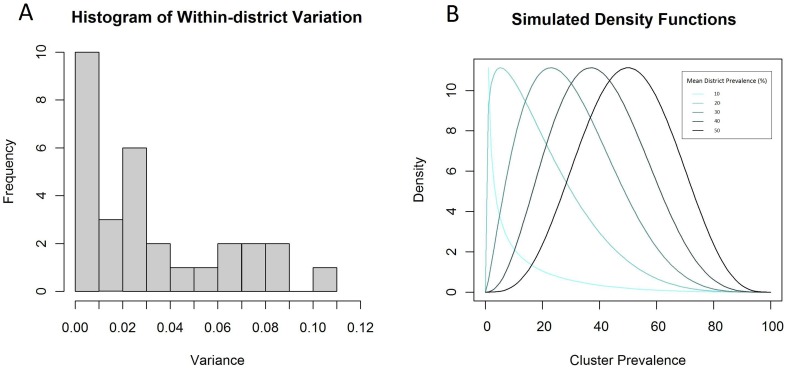
Histogram of the district variance of TF in Kenya (A) and density functions used to simulate data (B). Variance in the prevalence of active trachoma was quantified within 29 districts in Kenya. The mean within-district variance was then used to inform beta density functions for simulating cluster-level prevalence values for varying district level prevalence values.

Data on the prevalence of active trachoma were available for 305 clusters (non-overlapping sampling populations) from 29 districts in Kenya, surveyed as part of the National Trachoma Control Programme between 2004–2012 and included within the Global Atlas of Trachoma [23], [24]. These data represent available disaggregated data in a broadly similar context, and importantly include nearly all endemic districts. These data were used to model variation between and within districts (Figure 1) in order to inform simulation of realistic district and cluster-level prevalence values.

### Dataset expansion

The process of expanding the community dataset to simulate realistic data for 100 communities within each of 100 districts is fully described in the Technical Appendix (Supplement 1). In brief, district level prevalence estimates were generated covering all endemicity classes and used to simulate community level estimates of TF in children aged 1–9 years. The burden of TF within each simulated community was distributed among the population according to parameters initially defined by the above datasets (Table 3) in order to maintain disease clustering within households and subpopulations. Enrolment is defined as being “officially registered in a given educational programme, or stage or module thereof, regardless of age” [25], while attendance refers to an individual's presence at school at a given time. In these simulations we have assumed that all enrolled children attend on the day of the survey, however recognize that enrolment statistics are typically much higher than attendance. Enrolment was varied to assess the impact it has on sampling performance, and children identified as “school-going” were allowed to vary during the simulation process.

**Table 3:** Description of key epidemiological parameters used in the simulation model and sensitivity analysis^a^.

Key Parameter	Rationale	Method for estimation & Initial Value	Sensitivity Analysis
1. Age-specific prevalence of TF: TF in 1–5 years versus 6–9 years	In order to expand a cluster level prevalence estimate in children aged 1–9 years to the two age groups, need to know RR between groups. This will likely vary with endemicity.	Estimated from gold standard datasets Initial value: 2.0	Varied parameter: 1.3, 1.5, 1.8, 1.0, 2.0
2. Risk of TF in enrolled children vs non-enrolled children	Likely that enrolled children will have lower TF prevalence	Estimated from gold standard datasets Initial value: 0.5	Varied parameter: 0.25, 0.33, 0.5, 0.75, 1.0
3. School attendance	This will affect the sample size in schools of 6–15 year olds and affect the impact of parameter 2.	Ministry of Education data Initial value: 0.7	Varied parameter: 0.4 and 0.7
4. Clustering within households: risk of TF in children aged 1–5 years with a TF positive/negative sibling	Clustering at the household level will mean that children with TF positive siblings are more likely to have TF	Estimated from gold standard datasets Initial value: 0.2	Varied parameter: 0.1, 0.2, 0.3, 0.4, 0.5

TF: trachomatous inflammation–follicular; RR: relative risk.

a Random selection of 20 clusters were used in simulations for both.

To avoid basing simulations on data parametized by single village-level and district level datasets, additional pseudo-gold standard datasets were simulated varying each of the epidemiological parameters identified in Table 3 while holding other factors constant. This allowed an exploration of the impact of those parameters on the performance of the different sampling methodologies and the robustness of the different sampling approaches over other epidemiological settings. This included varying the level of household clustering quantified by the ICC, the relative risk of TF observed between enrolled and non-enrolled children, and the relative risk of TF between age group using parameters shown in Table 3.

### Sampling simulations

#### Survey methodologies

CRS for trachoma uses a standard two-stage or multi-stage design, often comprising a random selection of approximately 20 villages (clusters) at the first stage and selection of households at the second [26]. Selection of households may be carried out using simple random sampling, systematic sampling, the random walk or compact segment sampling. The sample size for CRS is calculated by defining parameters which include: expected prevalence estimates, acceptable error margin or precision, required confidence level, and design effect. In contrast, ITM employs convenience sampling of school children, pre-school children and women of child-bearing age to estimate the prevalence of trachoma [27]. At least two villages are selected per sub-district, with a minimum of 20 villages selected per district. In each village, a single school is randomly selected as the testing site. Children enrolled at that school are asked to come to the location, and adults from the community are also asked to assemble here and bring children aged 1–5 years. Systematic sampling is then used to select 25 children aged 1–5, 25 children aged 6–9 and 50 adult women (or 100 adults) aged ≥15 years.

#### Sampling process

A computerized simulation approach, using Monte Carlo methods, was used to randomly select 20 clusters from each district and sample individuals within each cluster according to the protocol for ITM and CRS (Table 2). For this analysis, a sample size of 100 individuals was assumed for CRS and participants selected from a random selection of households until the sample size met. It was assumed that children aged 1–5 years that would be brought to schools by their mother (or other adult household member) and sampled by ITM would be those with school-going siblings aged 6–9 years. We explored the impact of this assumption by also sampling a random selection of children in this age group. Sampling simulations were repeated 1000 times on each dataset using both methodologies.

### Analysis

District-level prevalence estimates generated by the two sampling methodologies were used to classify districts according to endemicity class for each simulation, using categories corresponding to established treatment thresholds: hypo-endemic (<10%), meso-endemic (10–30%) and hyper-endemic (>30%) (Table 1). The performance of each method was then quantified in terms of the proportion of times each district was correctly classified over 1000 simulations according to TF treatment thresholds.

### Operating Characteristic (OC) curve

Due to the complicated sampling distributions of these methodologies, it is not possible to calculate the full theoretical OC curves. However, we can visualize the empirical OC curves resulting from these simulation studies, which are generated from the proportion of times a district is correctly classified in each endemicity class using the two methodologies, over a “range” of district prevalence values. For each survey method, this allowed us to establish the range of district prevalence values in which the probability of correctly classifying a district is less than or equal to 0.80.

### Equivalency

Overall agreement in district endemicity classifications by the two methodologies was assessed using a weighted kappa-statistic. This statistic provides a measure of agreement between the two methods adjusted for chance, where a value of zero indicates agreement no better than chance. Weighting is useful when there are more than two ordered categories, so that the magnitude of disagreement between categories is allowed to vary (i.e., difference between <10% and 10–30% is not as great as that between <10% and >30%). Increasing kappa values correspond to better agreement between the two methods, where agreement is often interpreted as slight (<0.2), fair (0.2–0.4), moderate (0.4–0.6), substantial (0.6–0.8) and almost perfect (≥0.8) [28].

Equivalence between the two survey methods was formally evaluated by testing the null hypothesis that ITM yields the same programmatic results compared to CRS. The distribution of the difference in the proportion of correctly classified districts by ITM and CRS was generated and the mean and 95% CIs plotted in relation to delta, Δ, a threshold corresponding to a predefined level of difference deemed programmatically important. In these analyses, delta was initially assumed to be 20%, based on the rationale that this is equal to 80% of the simulations being classified the same by ITM and CRS and roughly corresponding to a standard level of acceptable error. Where the CI fell within this range, the survey methods were classified as equivalent for that district, while those that fell outside were classified as not equivalent and those that overlapped with the thresholds as inconclusive. Districts were stratified by the relative risk of TF and endemicity class to evaluate whether the equivalence of the two methodologies varied with these parameters.

## Results

### Estimated prevalence

**Figure 2:**
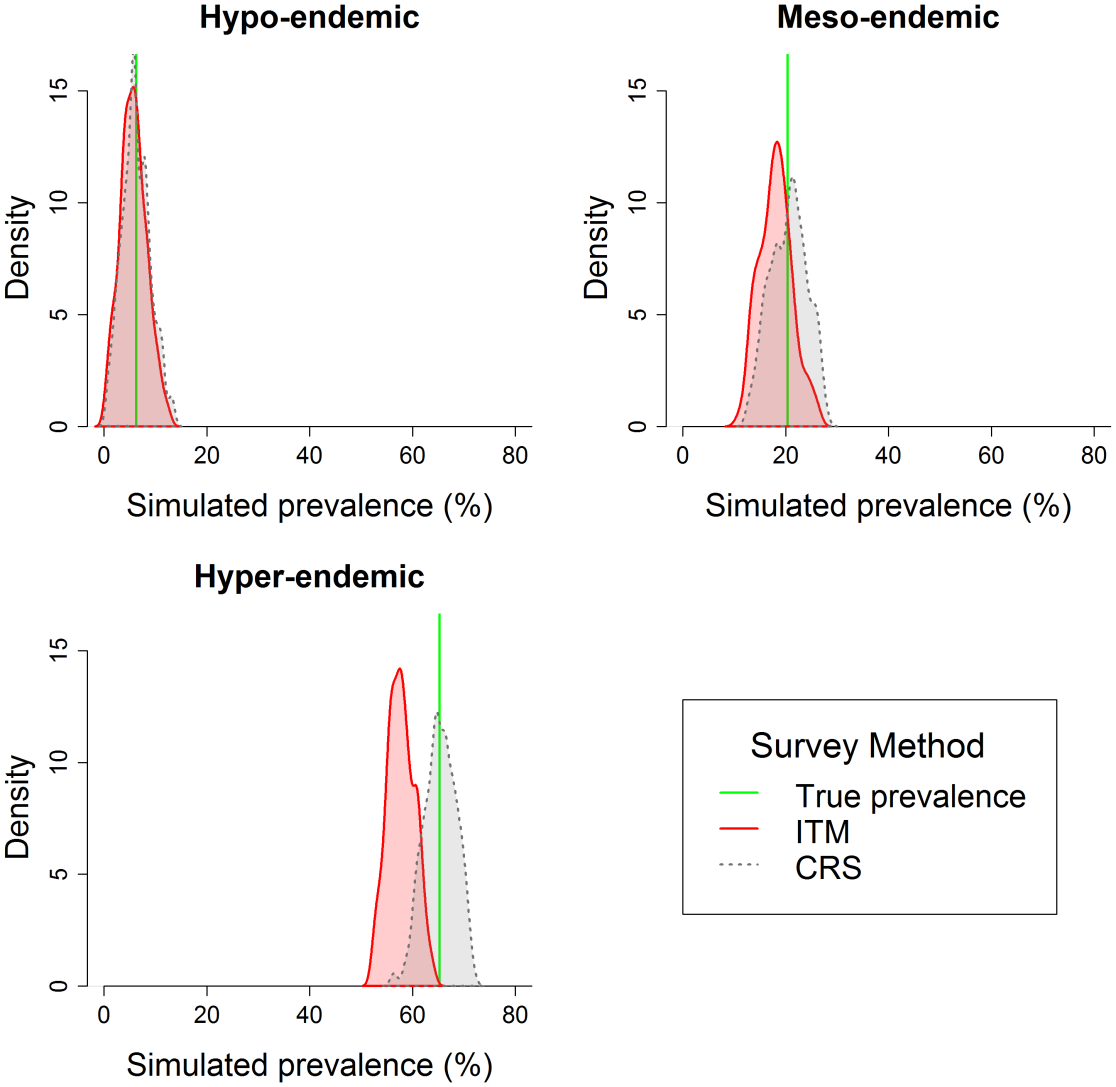
Density plots of prevalence estimates generated by CRS and ITM sampling methodologies. Plots are generated using simulated data and present results from a single district within each endemicity class. The red line represents the true district-level prevalence, the curves are histograms of values from 1000 simulations using the CRS method (red) and ITM method (blue).

**Figure 3:**
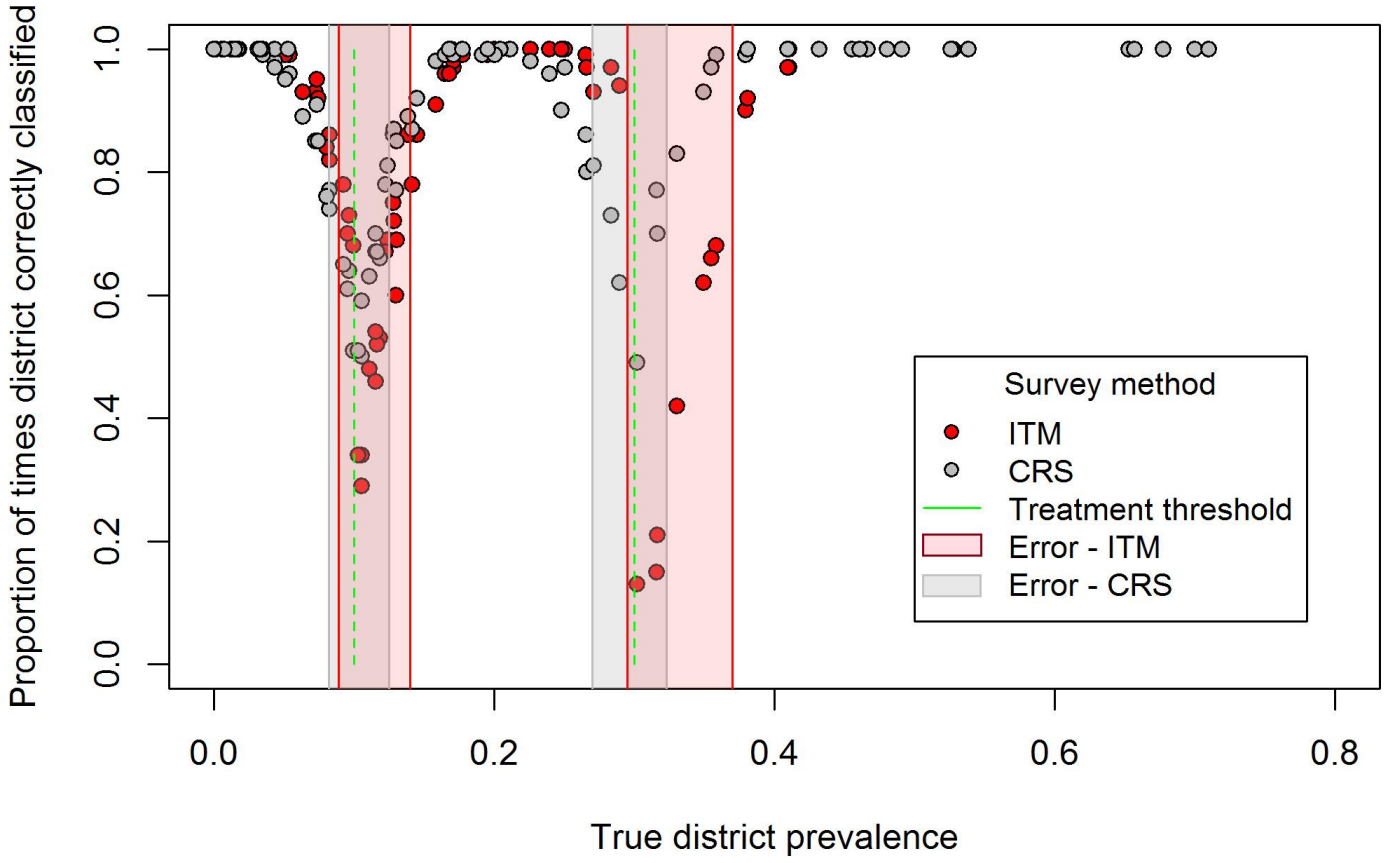
Performance of ITM and CRS compared to true prevalence. The proportion of times each of 100 districts were correctly classified by ITM and CRS were compared to true prevalence, where the relative risk of TF in enrolled and non-enrolled children is equal to 0.5 and enrolment rate is 0.7. The green lines correspond to the treatment thresholds and the boxes in red and grey around these thresholds to areas of “higher” misclassification, where the districts will be correctly classified less than 80% of the time.

Overall, the results indicate that ITM under-estimates the true prevalence of TF compared to CRS and that the magnitude of difference between estimates from these methodologies increases with endemicity. This is illustrated in Figures 2 and 3, which compare the two sampling strategies where all parameters are set to the initial values described in Table 3. Figure 2 presents filled density plots in example hypo-, meso-, and hyer-endemic districts, where the red line represents the true prevalence value for that district, the curves represent the distribution of prevalence estimates from the 1000 simulations using the CRS method (red) and ITM (blue). The results suggest that the systematic error resulting from school-based sampling is proportional to the prevalence, so that the absolute bias increases linearly as the prevalence increases.

### District-level classification

Figure 3 plots the proportion of times each of 100 districts were correctly classified (of 1000 simulations) against the district-level true prevalence for each sampling methodology, where the relative risk of TF in enrolled and non-enrolled children is equal to 0.5 and enrolment rate is 0.7. The green lines correspond to the treatment thresholds while the areas shaded red and grey around these thresholds have a “higher” risk of misclassification by the corresponding sampling methodology. Within these prevalence ranges, districts will be correctly classified less than 80% of the time. Performance of both CRS and ITM was lower closer to treatment thresholds. Compared to CRS, where misclassification error was fairly symmetrical around treatment thresholds, ITM tended to underestimate the prevalence of TF, resulting in a corresponding shift and widening of the region where potential error is known to be high.

Using a relative risk of TF in enrolled versus non-enrolled children equal to 0.5, there was “almost perfect” agreement (Kappa = 0.86) in district-level endemicity classification between ITM and CRS overall in the 1000 simulated samples. However, agreement between ITM and CRS decreased with increasing endemicity category, with substantial agreement found in hypoendemic districts (Kappa = 0.71) and only moderate agreement in mesoendemic (Kappa = 0.47) and hyperendemic districts (Kappa = 0.41).

**Figure 4:**
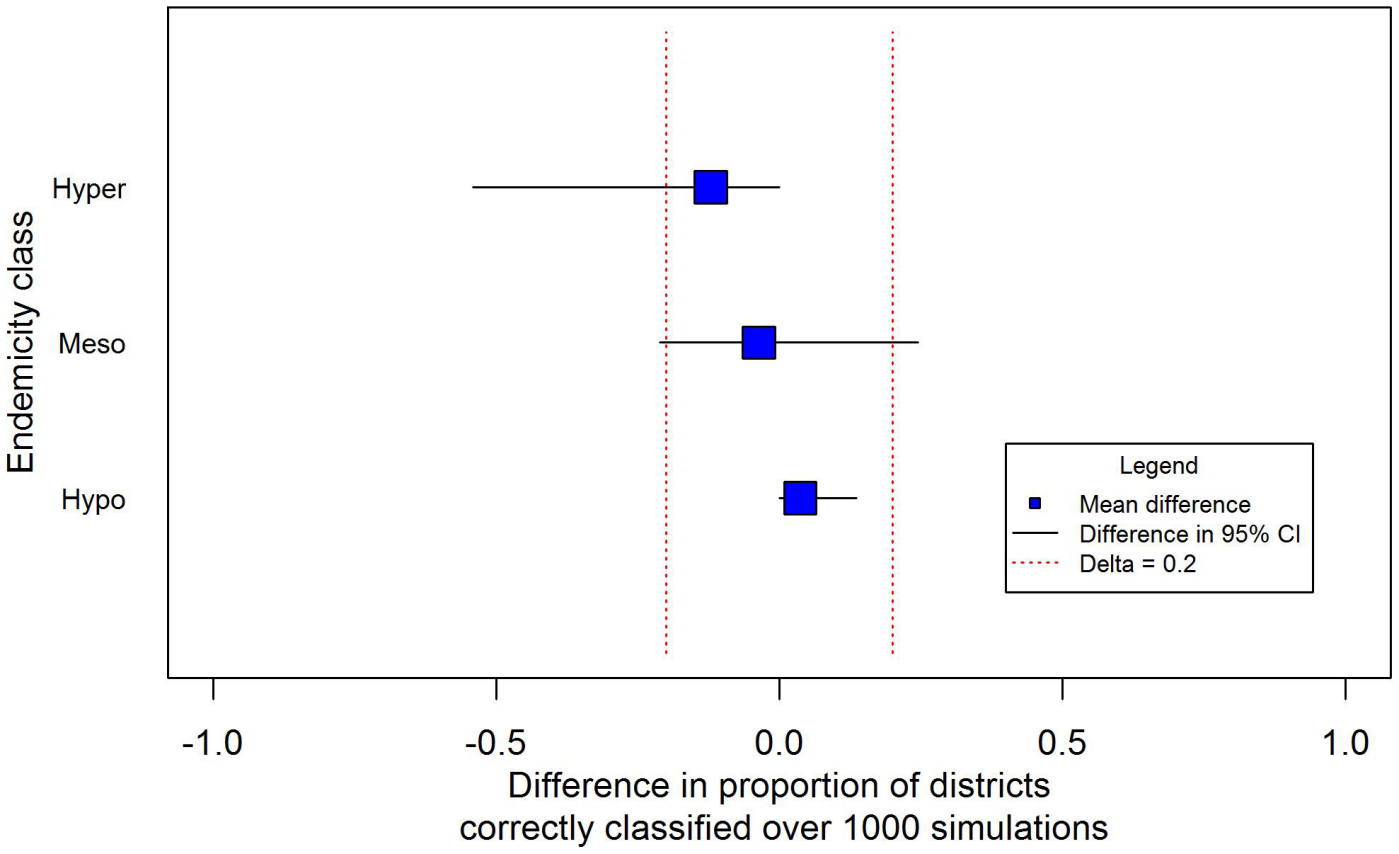
Equivalence of ITM compared to CRS by endemicity class. The figure presents the difference in the proportion of times ITM correctly classified districts compared to CRS (over 1000 simulations) by endemicity class in relation to an assumed value (20%) representing an important programmatic difference. The blue square is the mean difference in proportions and the lines correspond to the difference in the 95% CI. The two methods are deemed equivalent when ITM correctly classifies districts differently to CRS no more than 20% of the time.

The equivalence analysis in Figure 4 illustrates changes in the distribution of the difference in the proportion of correctly classified districts by ITM and CRS by endemicity class. The results suggest that the two sampling methodologies are equivalent in hypoendemic areas but the wider confidence intervals in meso- or hyper-endemic areas indicate that they less likely to be equivalent in these settings due to a greater degree of bias.

### Sensitivity analysis

**Figure 5:**
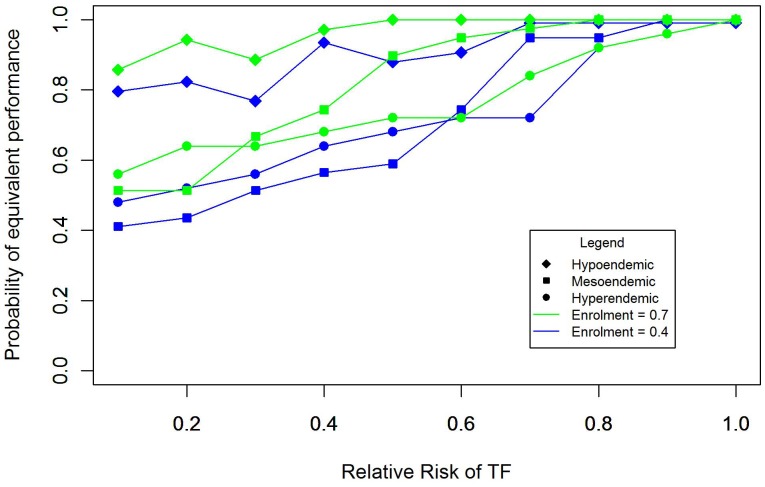
Equivalence in district classification by ITM and CRS. Equivalence is determined by calculating the difference in the probabilities that CRS and ITM will correctly classify a given district over 1000 simulations, and estimating whether this difference exceeds a delta equal to 0.2, signifying that two methods classify districts differently no more than 20% of the time. The figure presents equivalence by endemicity class and relative risk of TF in enrolled and non-enrolled children, where enrolment is equal to 0.4 (blue) or 0.7 (green).

**Figure 6:**
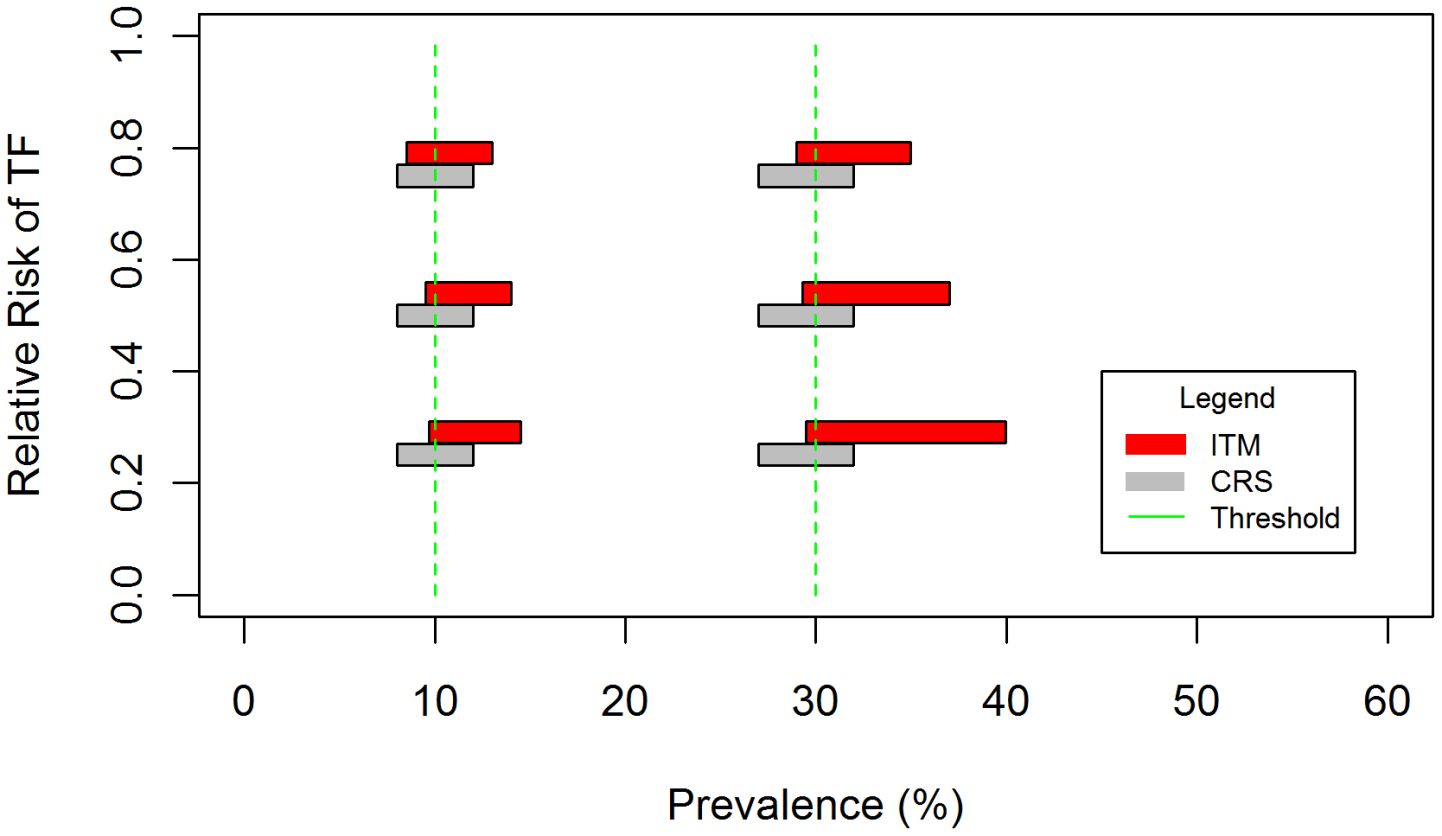
Range of true prevalence values with high risk of misclassification by CRS and ITM. Range of values in which the risk of misclassifying a district using CRS and ITM sampling methodologies is greater or equal to 0.20 around the 10% and 30% thresholds, with the enrolment rate equal to 0.7.

Sensitivity analysis of the impact of varying key parameters as shown in Table 3 suggested that the relative risk of TF between enrolled and non-enrolled children and the enrollment rate will define the performance of ITM. This is illustrated in figure 5 which plots the probability that ITM and CRS will give equivalent results in a district (i.e. the probabilities of correctly classifying a district using ITM and CRS differ no more than 0.20) given endemicity class and varying these parameters. Where enrollment is set as 0.7 and the relative risk is 0.75 or above, there is a high (≥80%) probability that ITM and CRS will be equivalent across all endemicity classes. As enrollment decreases and the difference in risk between enrolled and non-enrolled children increases, ITM increasingly misclassifies districts compared to CRS. This effect is likely to be greater in meso- and hyper- endemic districts, due to a greater magnitude of bias and resulting in misclassification over a wider range of prevalence values around the 10% and 30% thresholds. The impact on misclassification is also illustrated by Figure 6, which plots the range of prevalence values where the risk of misclassification using the two survey methodologies is greater or equal to 0.20. Classification error associated with CRS is symmetrically distributed approximately ±2 percent around each threshold and does not vary with these parameters. In contrast, the range of misclassification associated with ITM not only increases with a greater difference between enrolled and non-enrolled children, but also shifts to include more prevalence values above the threshold. Within this range of misclassification, the performance of ITM also decreases as a response to the degree of underestimation, so that in certain contexts ITM is unable to correctly classify any districts at or slightly above 30% prevalence. Variation in the relative risk of TF between age groups and the degree of household clustering defined by the ICC did not have an impact on performance. Evaluation of our assumption that children aged 1–5 years sampled by ITM were siblings of enrolled children also had no observable impact on the performance of ITM.

## Discussion

Our simulations show that over a range of epidemiological settings, ITM will under-estimate the true prevalence of TF. The error introduced by ITM also means that districts are more prone to misclassification according to treatment thresholds than by CRS. The extent of underestimation and misclassification of districts introduced by ITM is dependent on three main factors: (i) the district prevalence of TF; (ii) the relative risk of TF between enrolled and non-enrolled children within clusters; and (iii) the enrollment rate in schools. In general, the overall agreement between the two methods is high, but as the difference in risk of TF between enrolled and non-enrolled children becomes more pronounced, there is a shift in prevalence estimates corresponding to the magnitude of the bias. In these situations, the null hypothesis of programmatic equivalence between the two methodologies is not supported.

Use of a school-based platform is a key methodological difference between CRS and ITM and, while the potential pitfalls of this approach are well recognised, the impact of this strategy on treatment decisions has not been systematically evaluated until now [6], [29]. Our simulations highlighted the key influence of the relative risk of TF between enrolled and non-enrolled children and the enrollment rate on the performance of ITM. Furthermore, we were able to quantify the impact of these parameters on district classification over a range of endemicity settings. In areas where the risk of TF is similar between enrolled and non-enrolled children, there is evidence that CRS and ITM will be equivalent and classify districts correctly within an acceptable range of difference. Where risk is lower in enrolled children, a negative bias is introduced that is proportional to the magnitude of the difference in risk and reflected in greater absolute discrepancies between the two sampling methodologies as prevalence of TF increases. A lower enrolment rate effectively constrains the “sample” of the total population of children aged 6–9 attending schools and has the effect of increasing uncertainty around the prevalence estimate due to the greater effect of a positive child in the sample [30]. Compared to CRS, where misclassification error is fairly symmetrical around treatment thresholds across all scenarios, ITM can introduce a bias-dependent right shift and widen the range of prevalence values where misclassification error is high. In contrast, varying the relative risk of TF between age groups and the average ICC did not have a noticeable impact on performance of ITM and CRS at the district level, either in magnitude or shift.

As a consequence of this potential bias, ITM may be less likely than CRS to misclassify areas as greater than 10% or 30% when the true prevalence is below this threshold, but more likely to misclassify areas as lower when the true prevalence is higher. Misclassification is more comparable between the two methodologies at the 10% threshold, particularly when the relative risk between enrolled and non-enrolled children is closer to one. At this threshold, the misclassification by ITM would result in resources being allocated for further surveys at the subdistrict level instead of implementing MDA for the entire district. In practice, the difference in performance is most likely to impact interventions around the 30% threshold, where areas misclassified by ITM would be treated for three years before an impact survey instead of being treated for five years. Districts that fall within areas of high misclassification are of operational interest and the optimal choice of survey design is likely to be a function of the cost of the surveys, the costs of treatment associated with misclassification around both thresholds and the likely impact of treatment decisions on long term transmission dynamics. For example, while a particular survey design may be a cost-effective method to classify districts at a given round, a more accurate but more expensive survey design may allow quicker elimination of the disease leading to cost-savings in the future. Incorporating costs and the impact of treatment decisions on transmission was beyond the scope of this paper, but is the focus of future study.

Our use of computerised simulation has a number of advantages over field evaluations of trachoma sampling approaches [8], [16]. First, whereas inadequate evidence was available for meso- and hyperendemic settings, our approach allowed evaluation of ITM and CRS over a range of epidemiological settings. Second, simulations allowed the two sampling methodologies to be carried out independently of one another and repeated 1000 times for each district, thus accounting for sampling error in our estimates of performance. Finally, this approach allows key parameters to be explicitly defined and varied in a sensitivity analysis in order to explore their impact on performance in different contexts. This aspect of the study is important, as these parameters are likely to vary widely in settings where ITM might be used to generate TF prevalence estimates.

Although our study explored the performance of ITM and CRS in varying contexts, there are a number of potential limitations that may limit its generalisability. First, although key factors were varied in order to test sampling strategies in different epidemiological scenarios, exploring datasets similar to the data from Kahe in Tanzania and from Kenya would allow a more realistic range of parameters to be incorporated. In addition, parameterisation of the model assumed constant relationships which may be more complex in reality. Certain factors, like household clustering of trachoma, may vary markedly based on local transmission intensity, however no clear and consistent relationship was supported by available data. This may partly be due to random error introduced by the clinical sign TF, which is known to be an unreliable marker of *C. trachomatis* infection [31], [32]. A better estimation of these parameters, such as the relative risk of TF between enrolled and non-enrolled children, based on their relationship with endemicity may require collection of new data in the field. Second, these simulations sampled participants from a single demographic and household structure based on a community from Tanzania. Although the children selected as “enrolled” varied in the simulated datasets, it is possible that disease clustering within households might have a greater effect in other community structures. Furthermore, these simulations represent a general sampling scenario, and in the field there is more variation in the way that ITM and CRS are implemented. (For example, ITM randomly samples two clusters per subdistrict with a minimum of 20 per district, so the number of clusters sampled varies indirectly with district size [27]. In contrast, the number of clusters sampled by CRS is dependent on population size and is often selected using probability proportional to size in order to estimate a reliable district-level prevalence [6].) While use of a school-based survey platform offers a number of operational advantages, it is difficult to justify this approach in many contexts. In actual practice, one might expect trachoma “hotspots” to have poorer socioeconomic conditions and lower school enrollment, thus limiting the potential use of ITM to identify disease foci. More widespread collection of indicators of enrollment and attendance as part of trachoma surveys is encouraged in order to inform survey design. In addition, there is a lack of guidance on how ITM sampling methods would be operationalised in the event of non-response from family members bringing young children to the school. If the older children were oversampled, or a smaller sample of older children accepted, then ITM would underestimate the prevalence of TF to a greater degree. Finally, both the threshold of “acceptable difference” to be used in the equivalence analysis and the thresholds themselves deserve more discussion. To some degree, treatment thresholds are imprecise as they are based on unreliable clinical indicators and the impact on transmission of misclassifying a district that has a prevalence of 9% versus 12% is not well defined. As the elimination target for active trachoma is to reduce its prevalence to less than 5% in every sub-district, the transmission dynamics around these lower thresholds is of crucial interest. The degree of acceptable difference in performance between survey designs will depend on these transmission dynamics over the course of a control programme, as well as costs associated with misclassification.

The results from this study strengthen the evidence base around trachoma sampling methodologies and demonstrate the advantages of using a simulated approach to evaluate different sampling scenarios. To a large extent, the results from these simulations reflect a known limitation of school-based sampling: that resulting prevalence estimates are unreliable when the enrollment is low and/or the risk of disease in schools differs from communities. However, quantification of the performance of ITM at the district level in different contexts provides important information for national control programmes. In areas where enrolment is known to be very high, and it can be reliably inferred that the bias is minimized, then ITM may provide a rapid, cost-effective alternative to CRS [8], [33]. Future work could incorporate costing of different survey approaches and extension to include mathematical modeling to simulate the impact of different combinations of control interventions on transmission [34].

This paper serves as a demonstration of the use of sampling simulations to explore alternative sampling approaches not only for trachoma but also for other NTDs. We propose that this methodology be adopted as a cost-effective methodology to identify and evaluate potential strategies for the mapping, monitoring and evaluation, and surveillance, prior to field testing in multiple settings. Such simulations can identify key parameters including performance of sampling strategies and help inform the design of field evaluations. In turn, field studies can provide better estimates of key parameters and serve to refine simulation. We advocate an iterative process of simulation and field studies to identify optimal and cost-effective sampling strategies for a range of NTDs.

## Supporting Information

Appendix S1 Technical appendix.
(DOC)

## References

[1] WHO (2011). Report of the fifteenth meeting of the WHO alliance for the Global Elimination of Blinding Trachoma by 2020. Geneva: WHO.

[2] Deribe K, Meribo K, Gebre T, Hailu A, Ali A, et al (2012) The burden of neglected tropical diseases in Ethiopia, and opportunities for integrated control and elimination. Parasit Vectors 5: 240. 23095679 10.1186/1756-3305-5-240 PMC3551690

[3] Kabatereine NB, Malecela M, Lado M, Zaramba S, Amiel O, et al (2010) How to (or not to) integrate vertical programmes for the control of major neglected tropical diseases in sub-Saharan Africa. PLoS Negl Trop Dis 4: e755. 20614017 10.1371/journal.pntd.0000755 PMC2894133

[4] Koukounari A, Toure S, Donnelly CA, Ouedraogo A, Yoda B, et al (2011) Integrated monitoring and evaluation and environmental risk factors for urogenital schistosomiasis and active trachoma in Burkina Faso before preventative chemotherapy using sentinel sites. BMC Infect Dis 11: 191. 21749703 10.1186/1471-2334-11-191 PMC3161883

[5] Linehan M, Hanson C, Weaver A, Baker M, Kabore A, et al (2011) Integrated implementation of programs targeting neglected tropical diseases through preventive chemotherapy: proving the feasibility at national scale. Am J Trop Med Hyg 84: 5–14. 21212194 10.4269/ajtmh.2011.10-0411 PMC3005506

[6] Ngondi J, Reacher M, Matthews F, Brayne C, Emerson P (2009) Trachoma survey methods: a literature review. Bull World Health Organ 87: 143–151. 19274367 10.2471/BLT.07.046326 PMC2636192

[7] Brooker S, Kabatereine NB, Gyapong JO, Stothard JR, Utzinger J (2009) Rapid mapping of schistosomiasis and other neglected tropical diseases in the context of integrated control programmes in Africa. Parasitology 136: 1707–1718. 19450373 10.1017/S0031182009005940 PMC2777245

[8] Pelletreau S, Nyaku M, Dembele M, Sarr B, Budge P, et al (2011) The field-testing of a novel integrated mapping protocol for neglected tropical diseases. PLoS Negl Trop Dis 5: e1380. 22102921 10.1371/journal.pntd.0001380 PMC3216917

[9] Thylefors B, Dawson CR, Jones BR, West SK, Taylor HR (1987) A simple system for the assessment of trachoma and its complications. Bull World Health Organ 65: 477–483. 3500800 PMC2491032

[10] Mabey D, Solomon A, Foster A (2003) Trachoma. Lancet 362: 1736–1736. 10.1016/S0140-6736(03)13914-1 12885486

[11] Harding-Esch EM, Edwards T, Sillah A, Sarr I, Roberts CH, et al (2009) Active trachoma and ocular Chlamydia trachomatis infection in two Gambian regions: on course for elimination by 2020? PLoS Negl Trop Dis 3: e573. 20027217 10.1371/journal.pntd.0000573 PMC2791206

[12] King J, Odermatt P, Utzinger J, Ngondi J, Bamani S, et al Trachoma among children in community surveys from four African countries and implications of using school surveys for evaluating prevalence. International Health (under review).. 10.1093/inthealth/iht027 24179180

[13] Bailey R, Osmond C, Mabey DC, Whittle HC, Ward ME (1989) Analysis of the household distribution of trachoma in a Gambian village using a Monte Carlo simulation procedure. Int J Epidemiol 18: 944–951. 2621031 10.1093/ije/18.4.944

[14] Katz J, Zeger SL, Tielsch JM (1988) Village and household clustering of xerophthalmia and trachoma. Int J Epidemiol 17: 865–869. 3265700 10.1093/ije/17.4.865

[15] Polack SR, Solomon AW, Alexander ND, Massae PA, Safari S, et al (2005) The household distribution of trachoma in a Tanzanian village: an application of GIS to the study of trachoma. Trans R Soc Trop Med Hyg 99: 218–225. 15653125 10.1016/j.trstmh.2004.06.010 PMC6917506

[16] Dorkenoo AM, Bronzan RN, Ayena KD, Anthony G, Agbo YM, et al (2012) Nationwide integrated mapping of three neglected tropical diseases in Togo: countrywide implementation of a novel approach. Trop Med Int Health 17: 896–903. 22594642 10.1111/j.1365-3156.2012.03004.x

[17] Sturrock HJ, Gething PW, Clements AC, Brooker S (2010) Optimal survey designs for targeting chemotherapy against soil-transmitted helminths: effect of spatial heterogeneity and cost-efficiency of sampling. Am J Trop Med Hyg 82: 1079–1087. 20519603 10.4269/ajtmh.2010.09-0702 PMC2877414

[18] Sturrock HJW, Gething PW, Ashton RA, Kolaczinski JH, Kabatereine NB, et al (2011) Planning schistosomiasis control: investigation of alternative sampling strategies for Schistosoma mansoni to target mass drug administration of praziquantel in East Africa. International Health 3: 165–175. 24038366 10.1016/j.inhe.2011.06.002

[19] Edwards T, Smith J, Sturrock HJ, Kur LW, Sabasio A, et al (2012) Prevalence of trachoma in unity state, South Sudan: results from a large-scale population-based survey and potential implications for further surveys. PLoS Negl Trop Dis 6: e1585. 22506082 10.1371/journal.pntd.0001585 PMC3323519

[20] Olives C, Valadez JJ, Brooker SJ, Pagano M (2012) Multiple category-lot quality assurance sampling: a new classification system with application to schistosomiasis control. PLoS Negl Trop Dis 6: e1806. 22970333 10.1371/journal.pntd.0001806 PMC3435238

[21] Minetti A, Riera-Montes M, Nackers F, Roederer T, Koudika MH, et al (2012) Performance of small cluster surveys and the clustered LQAS design to estimate local-level vaccination coverage in Mali. Emerg Themes Epidemiol 9: 6. 23057445 10.1186/1742-7622-9-6 PMC3502089

[22] Pezzoli L, Andrews N, Ronveaux O (2010) Clustered lot quality assurance sampling to assess immunisation coverage: increasing rapidity and maintaining precision. Trop Med Int Health 15: 540–546. 20214765 10.1111/j.1365-3156.2010.02482.x

[23] Karimurio J, Gichangi M, Ilako DR, Adala HS, Kilima P (2006) Prevalence of trachoma in six districts of Kenya. East Afr Med J 83: 63–68. 16862999 10.4314/eamj.v83i4.9417

[24] Ministry of Health of Kenya (2010) District level prevalence estimates of trachoma in Kenya.

[25] UNESCO (United Nations Educational, Scientific, and Cultural Organization) (2011) International Standard Classification of Education (ISCED). Paris: United Nations Educational, Scientific and Cultural Organization.

[26] Solomon AW, Zondervan M, Kuper H, Buchan J, Mabey DC, et al.. (2006) Trachoma control - a guide for programme managers. Switzerland: World Health Organization.

[27] Mathieu E (2011) Field Manual for Integrated NTD Surveys. Atlanta, GA: Centers for Disease Control and Prevention,.

[28] Landis JR, Koch GG (1977) The measurement of observer agreement for categorical data. Biometrics 33: 159–174. 843571

[29] Dawson CR, Jones BR, Tarizzo ML, WHO (1981) Guide to trachoma control in programmes for the prevention of blindness: Geneva : World Health Organization.

[30] Jovani R, Tella JL (2006) Parasite prevalence and sample size: misconceptions and solutions. Trends Parasitol 22: 214–218. 16531119 10.1016/j.pt.2006.02.011

[31] Solomon AW, Peeling RW, Foster A, Mabey DC (2004) Diagnosis and assessment of trachoma. Clin Microbiol Rev 17: 982–1011, table of contents. 15489358 10.1128/CMR.17.4.982-1011.2004 PMC523557

[32] See CW, Alemayehu W, Melese M, Zhou Z, Porco TC, et al (2011) How reliable are tests for trachoma?–a latent class approach. Invest Ophthalmol Vis Sci 52: 6133–6137. 21685340 10.1167/iovs.11-7419 PMC3176003

[33] Brooker S, Kolaczinski JH, Gitonga CW, Noor AM, Snow RW (2009) The use of schools for malaria surveillance and programme evaluation in Africa. Malar J 8: 231. 19840372 10.1186/1475-2875-8-231 PMC2768743

[34] Lietman TM, Gebre T, Ayele B, Ray KJ, Maher MC, et al (2011) The epidemiological dynamics of infectious trachoma may facilitate elimination. Epidemics 3: 119–124. 21624783 10.1016/j.epidem.2011.03.004 PMC3869790

