# Supplementary material for: Comparing the Performance of Cluster Random Sampling and Integrated Threshold Mapping for Targeting Trachoma Control, Using Computer Simulation
Source: PLoS Negl Trop Dis. 2013 Aug 22;7(8):e2389. doi: 10.1371/journal.pntd.0002389 (PMC3749968; doi:10.1371/journal.pntd.0002389)
Supplement: Appendix S1 — Technical appendix. (DOC) [file pntd.0002389.s001.doc]

Technical Appendix

*Parameterisation & expansion of dataset*

Simulated data were parameterised using full census data from Kahe. The relative risk of TF between children aged 1-5 and 6-9 years was estimated (RR_age_ = 2.0) from the full dataset. Using data from individuals for which enrolment data were available (n=421) the relative risk of TF was estimated between enrolled and non-enrolled children aged 6-9 years (RR_enrol_ = 0.5).. In addition, an analysis of variance estimator was used to estimate the household intraclass correlation (ICC) present in Kahe, which was equal to 0.26 . Initially, the enrolment rate (R_enroll_) was set as 0.7 and assumed to not cluster within households. This assumption was based on results from a logistic regression model looking at the effects of these indicators on risk of TF in children aged 6-9 years. This model showed that individual level school attendance was the main factor in this context (odds ratio=0.52, p= 0.05), and having a school-going sibling was not associated with any additional risk (p=0.71). Similarly, in children 1-5 years old, having a school-going sibling was not associated with risk of TF after adjusting for household clustering (p=0.13).

Between and within district variation was estimated by fitting a beta distribution to the district-level and cluster-level TF prevalence data, respectively. The average variance and simulated mean prevalence were then used to define the sampling distributions for the two levels, with the mean of prevalences set as 0.2 at the district-level and equal to the simulated district prevalence for the cluster sampling distribution, from which cluster prevalence values were randomly drawn.

The dataset was subsetted, retaining data for the relevant age group (1-9 years). This dataset was then replicated to generate identical household and demographic structures for the specified number of communities (*n*) within each of *k* districts.

*Simulation of cluster data*

We outline a method in which individual disease status was then simulated for an *n* x *k* dataset using a methodology based on previous work for generating correlated binary random vectors outlined in Olives et al . This approach was modified to use household as a grouping factor and simulated data for each subgroup separately, so that each subgroup maintained household-level clustering, the specified RR_age_ and RR_enrol_ and summed to the overall cluster-level prevalence.

For each cluster within each district, the enrolment was simulated so that a specified proportion of children set by R_enroll_ were randomly selected and assigned as enrolled. TF cases were simulated by the two groups (age and enrolment) with the goal of ensuring the RR_age_ of TF in unenrolled and RR_enroll_ in 6-9s was preserved. To do so, we assumed that prevalence in enrolled 1-5s was zero (since we assume there are no 1-5s in school) and solved the following system of equations for the marginal prevalence of TF by age and enrolment:

1.
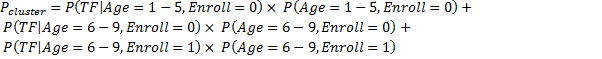

2.
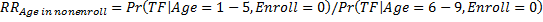

3.
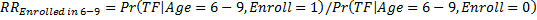


Once the marginal TF prevalences by age and enrolment were derived, the household ICC was introduced by using the methodology outlined in Olives et al [1].

**References**

1. Ridout MS, Demetrio CG, Firth D (1999) Estimating intraclass correlation for binary data. Biometrics 55: 137-148.

2. Olives C, Pagano M, Deitchler M, Hedt BL, Egge K, et al. (2009) Cluster designs to assess the prevalence of acute malnutrition by lot quality assurance sampling: a validation study by computer simulation. J R Stat Soc Ser A Stat Soc 172: 495-510.
